# Bacteriostatic and Antibiofilm Efficacy of a Nisin Z Solution against Co-Cultures of *Staphylococcus aureus* and *Pseudomonas aeruginosa* from Diabetic Foot Infections

**DOI:** 10.3390/life13020504

**Published:** 2023-02-11

**Authors:** Isa Serrano, Bernardo Alhinho, Eva Cunha, Luís Tavares, Alexandre Trindade, Manuela Oliveira

**Affiliations:** 1 CIISA—Center for Interdisciplinary Research in Animal Health, Faculty of Veterinary Medicine, University of Lisbon, Avenida da Universidade Técnica, 1300-477 Lisboa, Portugal; 2 Associate Laboratory for Animal and Veterinary Sciences (AL4AnimalS), 1300-477 Lisboa, Portugal; 3 Presently at Egas Moniz Center for Interdisciplinary Research, Egas Moniz School of Health and Science, 2829-511 Caparica, Portugal

**Keywords:** antibacterial, antibiofilm, bacteriostatic, diabetes mellitus, diabetic foot infections, EDTA, guar gum, Nisin Z, *Pseudomonas aeruginosa*, *Staphylococcus aureus*

## Abstract

Diabetes mellitus (DM) patients frequently develop diabetic foot ulcers (DFU) which are generally infected by a community of microorganisms, mainly *Staphylococcus aureus* and *Pseudomonas aeruginosa*. These bacteria exhibit a multi-drug resistance profile and biofilm-forming ability which represent a hurdle in the treatment of diabetic foot infections (DFI). We aimed to evaluate the potential of Nisin Z, an antimicrobial peptide (AMP), as an alternative treatment for severe DFI. Nisin Z shows antibacterial activity against Gram-positive and Gram-negative bacteria and an increased antibacterial effect against Gram-negatives when added to EDTA. As such, Minimum Inhibitory Concentration (MIC), Minimum Bactericidal Concentration (MBC), Minimum Biofilm Inhibitory Concentration (MBIC), and Minimum Biofilm Eradication Concentration (MBEC) were determined for Nisin Z, Nisin Z + EDTA (0.4%), and Nisin Z + EDTA incorporated into guar gum, in order to test its efficacy against *S. aureus* and *P. aeruginosa* isolated from the same DFU. Results showed that Nisin Z added to the chelation agent EDTA displayed higher antibacterial and bacteriostatic efficacy against mono and dual co-cultures of *S. aureus* and *P. aeruginosa*, and higher antibiofilm efficiency against monocultures. Nisin Z was moderately cytotoxic at 200 µg/mL. Prospect in vivo studies are needed to confirm the potential of Nisin Z supplemented with EDTA to be used as a complement to conventional antibiotic therapy for severe DFI.

## 1. Introduction

Diabetes mellitus (DM) is a lifelong chronic metabolic disease that affects about 537 million people worldwide, and the prevalence of which is increasing [1]. Approximately 30% of patients with diabetes develop diabetic foot ulcers (DFU) [2], because of a complex interaction of several pathophysiological factors affecting the vascular and immune systems and causing nerve damage, often affecting the legs and feet [3]. Around 40% of patients with DFU experience a recurrence within one year after the ulcer has healed, while nearly 60% suffer recurrence within three years, and 65% within five years [4]. The severe loss of skin protective barrier creates an opportunity for tissue colonization by bacteria, and 50% of diabetic patients with a DFU are estimated to develop diabetic foot infections (DFI) in their lifetime [2], which can be colonized by a polymicrobial community of opportunistic microorganisms [5]. DFI usually become chronic, resulting in a decrease in life quality, repeated hospitalizations, nontraumatic lower extremity amputation, higher morbidity, and premature mortality [6]. 

*Staphylococcus aureus* is the DFI predominant pathogen, being frequently found together with aerobic gram-negative rods such as *Pseudomonas aeruginosa* in chronic and more severe infections [5,7]. Both species belong to the ESKAPE group and are known for their multi-drug resistance profile towards commonly used antimicrobial agents, being classified as high and critical priority pathogens, respectively, in the WHO priority pathogens list for R&D of new antibiotics [8,9,10]. These bacteria can produce several virulence factors associated to wound infection chronicity, including biofilms [5,7,9]. Although there is a synergy between both species that benefits each other in co-infected wounds and may protect them from the inhibitory action of some antibiotics [11], it has been shown that *P. aeruginosa* exhibits conspicuous negative interactions with *S. aureus*, producing virulence factors that will affect *S. aureus* development [12,13,14]. 

Biofilms are the most common form of bacterial growth irreversibly attached to surfaces. They are slime-enclosed aggregates of sessile and interactive polymicrobial communities, within a self-produced matrix of extracellular polymeric substances composed of polysaccharides, proteins, and extracellular DNA [9,15,16]. These bacterial communities demonstrate high resistance to most antibacterial agents as well as to host defenses [9,15,16,17].

The standard treatment approach for DFI includes the physical removal of the biofilms via debridement, followed by wound cleansing with an antiseptic solution, most frequently with chlorhexidine [18], and antibiotic therapy [19]. As approximately a quarter of severe DFI cases are not successfully treated [19], new antibacterial treatments for DFI, including the use of different antimicrobials to prevent the formation of antibiotic resistance [20] and membrane-acting drugs such as antimicrobial peptides (AMP), are urgent.

AMP acts independently of the bacteria’s metabolic state, having antibiofilm properties. Their action is generally associated with size, charge, hydrophobicity, amphipathicity, and flexibility. Unlike antibiotics, bacteria have a low probability of developing resistance to AMP [21,22]. One of the most studied AMPs is Nisin, a 34 amino acid residue long cationic peptide lantibiotic bacteriocin mostly produced by *Lactococcus lactis* as part of its defense system [23]. Nisin interacts with the bacterial cell wall precursor lipid II, leading to pore formation, which is believed to cause rapid dissipation of transmembrane electrostatic potential, resulting in membrane permeabilization and rapid bacterial cell death [14,23,24]. As Nisin A acts predominantly against Gram-positive bacteria, it is not suited for the treatment of polymicrobial DFI, which may correspond to up to 68% of chronic DFI [9]. For this purpose, Nisin Z, the closest variant to Nisin A, differing only by a single amino acid residue at position 27 (asparagine instead of histidine) [24,25], seems to be more valuable. 

Nisin Z presents antibacterial activity against both Gram-positive and Gram-negative bacteria [24,26], and an increased antibacterial effect against Gram-negatives when added to EDTA [27]. EDTA removes magnesium and calcium ions from the outer cell wall of Gram-negative bacteria, thereby releasing up to 50% of the lipopolysaccharide and displaying phospholipids of the inner membrane, enhancing the efficacy of other antimicrobials [28]. 

As AMP can be degraded or inactivated before achieving its target, guar gum has been used for the delivery of these compounds. Guar gum is a plant-based, uncharged, and water-soluble polysaccharide named galactomannan, which is a polymer of d-galactose and d-mannose. Due to quick solubility in cold water, pH stability, film-forming, gelling properties, and biodegradability, it finds broad applications in industries [29], offering a safe and effective system for drug delivery by different administrative routes [30,31,32].

The study herein presented aimed to evaluate the antibacterial and antibiofilm efficacy of three Nisin Z solutions, to choose the most effective against *S. aureus* and *P. aeruginosa* before proceeding to preclinical trials. As such, the Minimum inhibitory concentration (MIC), Minimal bactericidal concentration (MBC), Minimum Biofilm Inhibitory Concentration (MBIC), and Minimum Biofilm Eradication Concentration (MBEC) of Nisin Z solutions (Nisin Z, Nisin Z + EDTA (0.4%), and Nisin Z + EDTA (0.4%) incorporated in guar gum 0.75% (*w*/*v*)) against mono and dual planktonic and biofilm cultures formed by *S. aureus* and *P. aeruginosa* DFI strains, previously characterized by us [33,34,35,36], were assessed. Nisin Z cytotoxicity was also evaluated.

## 2. Materials and Methods

### 2.1. Strains and Cultural Conditions

Biofilm-producing DFI strains *S. aureus* Z25.2 and *P. aeruginosa* Z25.1, co-isolated from the same diabetic foot ulcer in an epidemiological study on DFI microbiota conducted by us and fully characterized [33,34,35,36], were used. Reference strains *S. aureus* ATCC 29,213 and *P. aeruginosa* ATCC 27,853 were used as control strains. Before testing, strains were inoculated in Brain Heart Infusion (BHI) agar (VWR, Leuven, Belgium) and incubated at 37 °C, for 24 h. Bacterial suspensions in a sterile saline solution with 10^8^ CFU/mL were prepared from plate cultures and diluted in fresh BHI broth (VWR, Leuven, Belgium) to 10^7^ and 10^6^ CFU/mL.

### 2.2. Nisin Z Preparation 

Nisin Z and guar gum were prepared as described [31]. Stock solutions from ultrapure Nisin Z (≥95% purity, NISIN Z) (Handary, Brussels, Belgium) were prepared in milli-Q purified water (Sigma-Aldrich, Darmstadt, Germany), filtered using a 0.2 µm Millipore filter (VWR, Leuven, Belgium), and stored at 4 °C. 

A guar gum gel of 1.5% (*w*/*v*) was prepared by dissolving 0.75 g of guar gum (Sigma-Aldrich, Darmstadt, Germany) in 50 mL of sterile distilled water and heat sterilized by autoclave. An EDTA disodium salt (Sigma-Aldrich, Darmstadt, Germany) stock solution of 64 mg/mL was prepared by dissolving 6.4 g of EDTA in 100 mL of sterile distilled water and filtered.

A set of dilutions of Nisin Z were prepared, corresponding to the following concentrations: 0.1, 0.25, 0.5, 1, 2.5, 5, 10, 15, 25, 50, 100, 200, 400, 800, 1000, and 1250 µg/mL. The set of dilutions of Nisin Z were incorporated within the gel in a proportion of 1:1, followed by vortex homogenization, obtaining a final gel of 0.75% (*w*/*v*), as previously described by us [31]. EDTA was added to a final concentration of 0.4% (4000 µg/mL) (see results).

### 2.3. Minimum Inhibitory Concentration (MIC) and Minimal Bactericidal Concentration (MBC)

MIC and MBC of three Nisin Z solutions (Nisin Z, Nisin Z + EDTA (0.4%), and Nisin Z + EDTA (0.4%) + guar gum gel 0.75% (*w*/*v*)), towards *S. aureus* Z25.2 and *P. aeruginosa* Z25.1 DFI strains and controls strains, were determined as previously described [31]. 

MIC value of EDTA and Nisin Z solutions (tested concentrations ranging from 0.1 to 400 µg/mL) were determined by microtitre broth dilution method [37]. MIC was carried out in 96-well flat-bottomed polystyrene microtitre plates (VWR, Radnor, PA, USA). Briefly, 50 µL of each Nisin Z solution was inoculated onto each well of a 96-well plate, except for the positive (200 µL of bacterial suspension) and negative control wells (200 µL of broth medium). Then, all wells, except for positive and negative controls, were inoculated with 150 μL of the 10^7^ CFU/mL suspensions, as follows: plates 1/2/3—*S. aureus* suspension (DFI and reference strain); 4/5/6—*P. aeruginosa* suspension (DFI and reference strain); 7/8/9—*S. aureus* + *P. aeruginosa* dual-suspension (1:1) (dual DFI and dual reference strains). Microplates were incubated overnight at 37 °C, and MIC determined as the lowest concentration of each Nisin Z solution that visually inhibits microbial growth. 

MBC value was determined by inoculating 3 μL of the suspensions of the wells with no visual bacterial growth onto BHI agar plates, incubated at 37 °C, for 24 h. MBC was determined as the lowest concentration of each Nisin Z solution at which no colonies were formed.

Experiments were conducted in triplicate, and independent replicates were performed at least three times on different days. For each strain, nine results were obtained and analyzed.

MIC and MBC were also determined for EDTA (250 to 8000 µg/mL) towards *S. aureus* Z25.2 and *P. aeruginosa* Z25.1 DFI [33] and controls strains, using the previously described protocol.

### 2.4. Minimum Biofilm Inhibitory Concentration (MBIC) and Minimum Biofilm Eradication Concentration (MBEC)

MBIC and MBEC of Nisin Z, Nisin Z + EDTA (0.4%), and Nisin Z + EDTA (0.4%) + guar gum gel 0.75% (*w*/*v*), were determined using a modified version of the Calgary Biofilm Pin Lid Device [38]. Briefly, bacterial suspensions were prepared as before and diluted to 10^6^ CFU/mL in Tryptic Soy Broth medium (TSB) (VWR, Brussels, Belgium) supplemented with 0.25% (*w*/*v*) glucose (Merck, NJ, USA). Then, 200 μL of suspensions were placed in 96-well flat-bottomed polystyrene microtitre plates, covered with 96-peg polystyrene lids (Nunc, Thermo Fisher Scientific, Roskilde, Denmark) and statically incubated overnight at 37 °C to allow biofilm formation on pegs. Nine plates with DFI or reference strains were prepared for each determination, as in the previous MIC and MBC protocols. 

Afterwards, peg lids were rinsed 3 times in normal saline to remove planktonic bacteria, placed on new microplates with 150 µL of fresh TSB with 0.25% glucose holding a set of 50 μL of Nisin Z concentrations (0.1, 0.25, 0.5, 1, 2.5, 5, 10, 15, 25, 50, 100, 200, 400, 800, 1000, 1250 µg/mL), and incubated for 24 h at 37 °C without shaking. Positive (bacteria) and two negative controls (Nisin Z solution and medium), 200 µL each, were included. Then, peg lids were removed, and MBIC estimated visually as the lowest concentration of each Nisin Z solution that inhibits microbial biofilm growth [31].

For MBEC determination, peg lids were rinsed in PBS, placed in new microplates with 200 μL of fresh TSB with 0.25% glucose, and incubated in an ultrasound bath (1 min, 50 Hz) to disperse biofilms from the pegs’ surface. Then, peg lids were discarded, microplates covered with normal lids and incubated for 24 h, at 37 °C. MBEC value was determined as the minimum concentration of each Nisin Z solution causing no visual growth relative to the positive control [15].

Experiments were conducted in triplicate, and independent replicates were performed at least three times on different days. For each strain, nine results were obtained and analyzed.

### 2.5. Cytotoxicity Assay 

Cytotoxicity assay was performed as previously described [32]. Adult Human primary adherent Epidermal Keratinocytes (HEKa) (PCS-200-011, ATCC, Manassas, VA, USA) were cultured according to the manufacturer’s instructions to assess the cytotoxic potential of each Nisin Z solution against eukaryotic cells. 

Cells were cultured in 75 cm^2^ cell culture flasks (Nunc, Thermo Fisher Scientific, Roskilde, Denmark) in Dermal Cell Basal Medium (ATCC, Manassas, VA, USA) supplemented with the Keratinocyte Growth Kit (ATCC, Manassas, VA, USA), and incubated at 37 °C in a 5% CO_2_ humidified atmosphere. After reaching a confluence of about 80%, cells were collected with trypsin-EDTA (0.25%, Gibco, Thermo Fisher Scientific, Roskilde, Denmark), diluted, and counted using a Neubauer hemocytometer.

HEKa cells (5000–10,000 cells per well) were seeded in flat bottom polystyrene 96-well microplates and incubated for 48 h at 37 °C, in a 5% CO_2_ humidified atmosphere. Afterwards, 180 µL of fresh medium was added, and HEKa cells incubated with 20 µL Nisin Z solutions in different Nisin Z final concentrations (5, 10, 15, 25, 50, 100, 200, 400, and 1250 µg/mL), for 24 h at 37 °C in a 5% CO_2_ humidified atmosphere. Doxorubicin hydrochloride (DOXO) 4 µM (Medac Gmbh, Wedemark, Germany) was used as positive control, and water, EDTA (0.4%) and 0.75% guar gum biogel as negative control.

Cell viability was determined using (3-(4,5-dimethylthiazol-2-yl)-2,5-diphenyltetrazolium bromide) tetrazolium reduction assay (MTT assay) kit (ab211091, Abcam, Cambridge, UK), with small changes to the manufacturer’s instructions. Briefly, growth medium was removed from all wells, and growth medium and MTT reagent (1:1) were added into each well. Cells were then incubated at 37 °C for 3 h, after which 150 µL of MTT solvent was added into each well to dissolve the formazan crystals. Microplates were wrapped in foil and agitated on an orbital shaker for 15 min at room temperature. Cell viability was evaluated using a microplate reader (BGM LABTECH, Ortenberg, Germany) to measure optical density at a wavelength of 584 nm. Blank control was the growth medium without cells. Cell viability was calculated as a percentage of the untreated control (growth media plus HEKa cells), which was assumed to be 100% viable.

Experiments were conducted in triplicate, and independent replicates were performed at least three times on different days. For each strain, nine results were obtained and analyzed.

### 2.6. Statistical Analysis

Data were analyzed using GraphPad Prism software for Windows version 9.4.1 (graph Pad, USA) and shown as mean ± standard error of mean (SEM). One-way ANOVA (*p* < 0.0001) and Tukey’s multiple comparisons test were performed. MIC, MBC, MBIC and MBEC analysis were conducted separately. In each of them, the differences between Nisin Z solutions were analyzed collectively for *S. aureus* Z.25.2, *P. aeruginosa* Z.25.1 and dual DFI co-culture. Regarding the DFI and control strains comparisons, the differences between Nisin Z solutions were analyzed for each DFI and control pair.

## 3. Results

### 3.1. MIC and MBC

**Figure 1:**
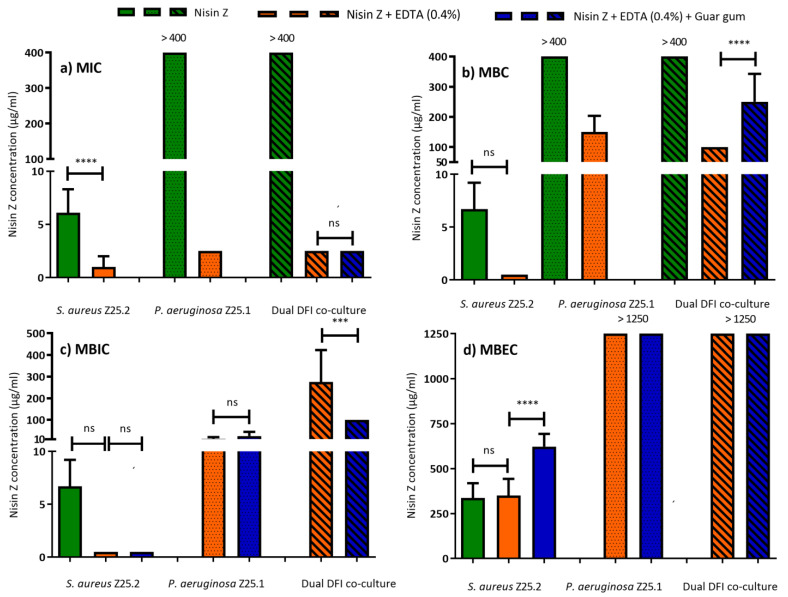
Minimum Inhibitory Concentration (MIC) (**a**), Minimal Bactericidal Concentration (MBC) (**b**), Minimum Biofilm Inhibitory Concentration (MBIC) (**c**), and Minimum Biofilm Eradication Concentration (MBEC) (**d**), of Nisin Z solutions, against *S. aureus* Z.25.2, *P. aeruginosa* Z.25.1, and dual co-cultures from diabetic foot infections (DFI). Data shown as means ± SEM; each group value is an average of mostly nine independent measurements. ns not significant: *** *p* < 0.001; **** *p* < 0.0001; One-way ANOVA, Tukey’s multiple comparison test.

MIC and MBC of EDTA were evaluated and showed no inhibitory effect in any tested concentration (250 to 8000 µg/mL) towards planktonic DFI and control strains. EDTA exhibited a MIC and MBC mean value of >8000 ± 0 µg/mL. Therefore, a final concentration of EDTA of 4000 of µg/mL was chosen to be added to Nisin Z solution. *S. aureus* Z25.2 was susceptible to low Nisin Z concentrations, but *P. aeruginosa* Z25.1 and dual DFI co-cultures were resistant to the antimicrobial peptide (MIC > 400 µg/mL). Nisin Z MIC towards *S. aureus* Z25.2 ranged from 5 to 10 µg/mL, with a mean value of 6.11 ± 2.2 µg/mL, and MBC values were two to 2.5-fold higher than MIC (MBC mean of 18.8 ± 6.9 µg/mL, ranging from 10 to 25 µg/mL). *P. aeruginosa* Z25.1 and the dual DFI co-culture were not affected by Nisin Z (MIC and MBC higher than 400 µg/mL) (Figure 1a,b).

When EDTA (0.4%) was added to Nisin Z, all suspensions were susceptible at low MIC values, with these results being statistically different when compared to Nisin Z MIC towards *S. aureus* Z.25.2 (Figure 1a). In addition, MBC values decreased and remained under 200 µg/mL (Figure 1b). Nisin Z MIC regarding *S. aureus* Z25.2 ranged between 0.25 and 2.5 µg/mL, with a mean value of 1.0 ± 1.1 µg/mL. MBC was four- to six-fold higher, with a mean of 8.6 ± 5.3 µg/mL, ranging between 1 and 15 µg/mL.

Nisin Z MIC regarding *P. aeruginosa* Z25.1 showed a mean value of 2.5 ± 0 µg/mL. MBC was 40 to 80-fold higher, with a mean value of 150.0 ± 53.5 µg/mL, ranging from 100 to 200 µg/mL. Nisin Z MIC regarding dual DFI co-culture showed a mean value of 2.5 ± 0 µg/mL, and MBC was 40-fold higher, with a mean value of 100.0 ± 0. 

After the addition of guar gum, only dual co-cultures were evaluated because it was anticipated that MIC and MBC values regarding DFI monocultures of *P. aeruginosa* Z.25.1 and *S. aureus* Z.25.2 would be similar than those for Nisin Z + EDTA (0.4%), and priority was given to the dual co-culture, as the more severe forms of DFI are commonly polymicrobial. The addition of guar gum did not statistically change Nisin Z MIC (2.5 µg/mL) regarding dual DFI co-cultures (Figure 1a), but it changed (*p* < 0.05) the MBC mean value to 250.0 ± 92.6 µg/mL (200 to 400 µg/mL) (Figure 1b).

Comparing the results for *S. aureus* Z.25.2 to those for the reference strain, only the difference in the Nisin Z MBC value was not significantly different. Comparing the results for *P. aeruginosa* Z.25.1 to those for the reference strain, only Nisin Z + EDTA (0.4%) MIC value was significantly different. Comparing the results from the dual DFI co-culture to those from the dual control, only Nisin Z + EDTA (0.4%) MIC and MBC values were significantly different (Table 1).

### 3.2. MBIC and MBEC

The biofilm mode of growth of the infecting organisms can impair DFI healing, since biofilm can resist antibiotic concentrations 10 to 10,000 times higher than those needed to eliminate planktonic forms [39]. Therefore, to evaluate MBIC and MBEC, Nisin Z was tested using concentrations up to 1250 µg/mL. As *P. aeruginosa* Z25.1 and dual DFI co-cultures were resistant to Nisin Z, it was assumed that they would maintain their resistant phenotype, therefore Nisin Z MBIC and MBEC towards these biofilms were not evaluated.

*S. aureus* Z.25.2 biofilm was susceptible to Nisin Z solutions at low concentrations, with MBIC ranging from 5 to 10 µg/mL, with a mean value of 6.7 ± 2.5 µg/mL, and MBEC ranging from 200 to 400 µg/mL, with a mean value of 366.7 ± 81.6 µg/mL. 

Nisin Z MBIC values towards *S. aureus* Z25.2 and *P. aeruginosa* Z.25.1 biofilms were low. The addition of EDTA (0.4%) to Nisin Z and of guar gum did not statistically change MBIC values towards *S. aureus* Z.25.2 biofilms (mean of 0.5 ± 0 µg/mL), but it changed MBEC values (*p* < 0.05), which remained under 1250 µg/mL, ranging from 200 to 400 µg/mL (mean of 350.0 ± 92.6 µg/mL) for Nisin + EDTA (0.4%), and from 400 to 800 µg/mL (mean of 622.2 ± 210.8 µg/mL) for Nisin Z + EDTA (0.4%) + guar gum (Figure 1c,d). 

Regarding *P. aeruginosa* Z.25.1 biofilms, the addition of guar gum did not statistically change MBIC, which increased from 12.5 ± 7.1 to 25.0 ± 18.9 µg/mL. MBEC remained above 1250 µg/mL (Figure 1c,d).

Dual DFI co-culture biofilms were susceptible to higher concentrations of Nisin Z + EDTA (0.4%). The addition of guar gum statistically decreased (*p* < 0.05) MBIC values regarding dual DFI co-culture from 275.0 ± 147.5 to 100 ± 0.0 µg/mL. MBEC remained above 1250 µg/mL (Figure 1c,d).

When comparing MBIC and MBEC values for *S. aureus* Z.25.2 to those for the reference strain, MBIC was statistically different only for Nisin Z, and MBEC for Nisin Z and Nisin Z + EDTA (0.4%). Comparing MBIC and MBEC values for *P. aeruginosa* Z25.1 to those for the reference strain, MBIC was statistically different in both solutions tested, and while MBEC value of Nisin Z + EDTA (0.4%) was 1007.1 ± 130.5 towards the reference strain, it was of > 1250 for *P. aeruginosa* Z25.1. Comparing MBIC values for dual DFI to dual control biofilms, they were not statistically different, and MBEC was >1250 µg/mL in both cases (Table 1). 

### 3.3. Cell Toxicity

Pursuant to ISO 10993-5, percentages of cell viability above 80% are considered as non-cytotoxicity, within 80–60% as weak, 60–40% as moderate, and below 40% as strong cytotoxicity [40]. Therefore, Nisin Z was non-cytotoxic from 5 µg/mL (HEKa cell viability of 90.3%) to 25 µg/mL (HEKa cell viability of 84.6%), weakly cytotoxic at 50 and 100 µg/mL (HEKa cell viability of 78.9% and 68.2%), moderately cytotoxic at 200 µg/mL (HEKa cell viability of 56.7%), and strongly cytotoxic at 400 and 1250 µg/mL (HEKa cell viability of 2.5 and 0.9%, respectively).

The addition of EDTA (0.4%), and of EDTA (0.4%) + guar gum gel 0.75% (*w*/*v*) to Nisin Z render these solutions cytotoxic in all concentrations tested, with cell viability ranging from 9.2% to 33.1% and from 4.6% to 17.4%, respectively (Table 2).

## 4. Discussion

The antibacterial activity of the Nisin Z suspension was improved by the addition of EDTA (0.4%) to this antimicrobial peptide, as *S. aureus* Z.25.2, *P. aeruginosa* Z.25.1 and dual DFI co-cultures were considered susceptible at low MIC values. These results are in accordance with a previous study in which the antimicrobial potential of Nisin A was analyzed against 23 *S. aureus* DFI biofilm producing isolates [31]. In that study, *S. aureus* Z.25.2 was considered susceptible to Nisin A at MIC = 100 µg/mL, a higher value than the one obtained in the present study (MIC of Nisin Z = 6.1 ± 2.2 µg/mL). The differences between the structure of the two molecules may explain the different results [24,25].

The addition of EDTA (0.4%) to Nisin Z resulted in a suspension with bactericidal potential against *P. aeruginosa* Z.25.1 and dual DFI co-cultures. Nisin Z solutions, with or without EDTA, had bactericidal potential against *S. aureus* Z.25.2 with low MBC values. In a previous study, the mean MBC value of Nisin A against *S. aureus* Z.25.2 was 600 µg/mL [31], a much higher value than the one obtained in the present study (MBC of Nisin Z = 18.8 ± 6.9 µg/mL). These differences observed in MIC and MBC values of Nisin A and Nisin Z against *S. aureus* Z.25.2 highlight the superior antibacterial efficacy of Nisin Z over Nisin A towards DFI isolates.

Results showed that the addition of guar gum to Nisin + EDTA (0.4%) did not statistically affect the suspension’s antibacterial efficacy, but it was detrimental to its bactericidal potential towards co-cultures.

Since antimicrobial agents are usually classified as bactericidal if the MBC is no more four times the MIC value [41], our results showed that Nisin Z is bactericidal against *S. aureus*, and Nisin Z with EDTA is bacteriostatic against *S. aureus* Z.25.2 and *P. aeruginosa* Z.25.1 in monocultures and dual co-cultures. However, Nisin Z + EDTA (0.4%) should be considered a valued AMP to kill planktonic *S. aureus*, as MBC (four to six-fold) was near the limit value to be considered bactericidal [41].

Nisin Z solutions showed high antibiofilm activity against *S. aureus* Z.25.2 and *P. aeruginosa* Z.25.1 biofilms, associated with low MBIC values. The addition of guar gum was beneficial for dual DFI co-cultures because it significantly increased the antibiofilm efficacy to nontoxic Nisin Z levels. Biofilm eradication was only attained towards *S. aureus* Z.25.2 biofilms, but still in concentrations over 200 µg/mL, and significantly more effective without adding guar gum.

Antibiofilm and eradication efficacy of Nisin Z was demonstrated to be superior to that of Nisin A, since results from a previous study showed that Nisin A MBIC value against *S. aureus* Z.25.2 was 40 µg mL and MBEC values > 1000 µg mL [31]. The difference in a single amino acid residue at position 27 (asparagine instead of histidine) [24,25], between Nisin A and its closest variant Nisin Z is enough for a higher antibacterial, antibiofilm and eradication potential against DFI *S. aureus*.

Regarding dual co-cultures, it is recognized that there is a synergy between both *P. aeruginosa* and *S. aureus* that may protect them from the inhibitory action of antibiotics [11]. If the synergy effects were stronger than the also known negative interactions of *P. aeruginosa* against *S. aureus*, in which *P. aeruginosa* produces anti-staphylococcal products and proteases causing *S. aureus* biofilm dispersion and cell lysis [12,42], dual co-cultures would show higher resistance to Nisin Z solutions than monocultures. This was not the case, since Nisin Z MIC, MBC, and MBEC towards dual DFI co-cultures were more similar to those towards *P. aeruginosa* Z.25.1 and generally higher than those towards *S. aureus* Z.25.2. Therefore, results suggest that, in vitro, the harmful action of *P. aeruginosa* against *S. aureus* overlaps the synergistic effect of being together.

Studies available on the cytotoxicity of Nisin regarding keratinocyte cells are limited, and results depend on the cell type. It was shown that Nisin Z, a naturally occurring variant of Nisin, does not induce apoptosis in human oral keratinocytes [43], and in another study showed that Nisin A concentrations up to 50 µg/mL were non-cytotoxic to HEKa cells [32]. 

According to [44], generally it can be said that if a compound is non-cytotoxic, then it will be tolerable in vivo. If the compound is moderately cytotoxic, there is a likelihood that the different cell types of an organ can effectively recover from the cytotoxic damage, or the damage will only be minimal [44]. In the present study, Nisin Z at 200 µg/mL was moderately cytotoxic to HEKa cells, meaning that it is within acceptable values for pharmaceutical/medical use; however, in vivo safeness must be further evaluated. Although the addition of EDTA (0.4%) to Nisin Z renders this solution cytotoxic to HEKa cells, EDTA concentration tested was within the range commonly used in therapeutical protocols and considered to be safe [45,46,47]. In fact, according to [47], there are two commercially available wound care products that contain EDTA: RescuDerm (NociPharm, Inc., Toronto, ON, Canada), and Biostep (Smith & Nephew Wound Management, Inc., Largo, FL, USA), both FDA approved. Moreover, EDTA is the medically-accepted FDA-approved treatment for lead poisoning in adults and children [48], and used in the treatment of band keratopathy, a degenerative condition of the cornea [46], through chelation therapy [46,48]. Therefore, EDTA cytotoxicity must be analyzed carefully, as it may be associated with specific in vitro conditions, and probably does not relate to an in vivo cell toxicity, and further analysis is needed. The detected cytotoxicity of Nisin Z with EDTA (0.4%) incorporated into guar gum was most possibly due to the presence of EDTA, as guar gum was considered non-cytotoxic in a previous study in which the incorporation of Nisin A into guar gum gel 0.75% (*w*/*v*) had no impact on Nisin A cytotoxicity [32].

## 5. Conclusions

Considering that Nisin Z showed acceptable values for pharmaceutical/medical use, and that EDTA concentration was within the range found in commercialized and safe products [45,46], it can be concluded that Nisin Z supplemented with EDTA (0.4%) is, among the tested solutions, the most promising to be used as a treatment for severe diabetic foot infections, considering that it showed: 

(1) Higher antibacterial efficiency (*S. aureus* Z.25.2 was considered susceptible at MIC ≥ 1.0 ± 1.1µg/mL, *P. aeruginosa* Z.25.1 and dual DFI co-culture at MIC ≥ 2.5 µg/mL);

(2) Higher bacteriostatic efficacy (*S. aureus* Z.25.2 was killed at MBC ≥ 8.6 ± 5.3 µg/mL, *P. aeruginosa* Z.25.1 at MBC ≥ 150.0 ± 53.5 µg/mL, and dual DFI co-culture at MBC ≥ 100 µg/mL); 

(3) Higher antibiofilm efficiency against monocultures (*S. aureus* Z.25.2 MBIC was ≥0.5 µg/mL, and *P. aeruginosa* Z.25.1 MBIC was ≥12.5 ± 7.1 µg/mL). Antibiofilm activity against dual DFI co-cultures was only possible at cytotoxic Nisin Z levels. 

Biofilm eradication was not attainable in any tested Nisin Z solution at moderate-cytotoxic levels. Nevertheless, this study highlights the antibacterial and antibiofilm activity of Nisin Z supplemented with EDTA against *S. aureus* and *P. aeruginosa*, which are ESKAPE pathogens commonly found colonizing diabetic foot ulcers. Further in vivo studies aiming at confirming and characterizing the solution antimicrobial activity towards polymicrobial diabetic foot infections are urgent. We expect that, in the future, Nisin Z supplemented with EDTA would be used as a safe and effective complement to antibiotics for the successful treatment of severe diabetic foot infected ulcers. It would ultimately contribute to stopping the diabetic foot infection from becoming chronic and limiting the quality of patients’ life.

## Figures and Tables

**Table 1:** Minimum Inhibitory Concentration (MIC), Minimal Bactericidal Concentration (MBC), Minimum Biofilm Inhibitory Concentration (MBIC) and Minimum Biofilm Eradication Concentration (MBEC) of Nisin Z solutions, against reference strains of *S. aureus*, *P. aeruginosa*, and dual suspension of both reference strains. Data shown as means ±SEM; each group value is an average of mostly nine independent measurements. ns not significant: * *p* < 0.1; ** *p* < 0.01; *** *p* < 0.001; **** *p* < 0.0001 versus diabetic foot infections (DFI) strain or dual DFI co-culture; One-way ANOVA, Tukey’s multiple comparison test.

	Nisin Z				Nisin Z + EDTA (0.4%)				Nisin Z + EDTA + Guar Gum	
Strains	MIC	MBC	MBIC	MBEC	MIC	MBC	MBIC	MBEC	MBIC	MBEC
*S. aureus* ATCC	10.0 ± 3.8 **	25.6 ± 10.8 ns	25.0 ± 0 ****	921.4 ± 172.9 ***	5 ± 0 **	83.5 ± 25.0 ****	0.5 ± 0.0 ns	725.0 ± 212.1 **	0.5 ± 0.0 ns	629.9 ± 316.8 ns
*P. aeruginosa* ATCC	>400	>400			50.0 ± 0 ****	114.3 ± 37.8 ns	61.1 ± 30.9 *	1007.1 ± 130.5	133.3 ± 50.0 ****	>1250
Dual suspension ATCC	>400	>400			50 ± 0 ****	212.5 ± 124.6 *	242.9 ± 151.2 ns	>1250	150.0 ± 53.5 ns	>1250

**Table 2:** Cell viability (percentage) of adult human primary adherent epidermal keratinocytes (HEKa) when treated with Nisin Z solutions. Each group value is an average of nine independent measurements.

	Nisin Z Concentrations (µg/mL)									Positive Control	Negative Control		
	5	10	15	25	50	100	200	400	1250	DOXO	H_2_O	EDTA (0.4%)	Guar Gum 0.75% (*w*/*v*)
Nisin Z	90.3	88.0	85.8	84.6	78.9	68.2	56.7	2.5	0.9	9.7	87.9		
Nisin Z + EDTA (0.4%)	33.1	17.4	11.7	11.1	24.3	15.6	9.6	4.3	9.2	16.0	93.3	16.5	
Nisin Z + EDTA (0.4%) + guar gum 0.75% (*w*/*v*)	17.4	9.7	8.1	5.0	12.0	10.5	9.9	9.5	4.6	12.5	77.3	21.0	92.2

## Data Availability

The data presented in this study are available on request from the corresponding author. The data are not publicly available due to privacy reasons.

## References

[1] Magliano D.J., Boyko E.J. (2021). IDF Diabetes Atlas.

[2] Chastain C.A., Klopfenstein N., Serezani C.H., Aronoff D.M. (2019). A Clinical Review of Diabetic Foot Infections. Clin. Podiatr. Med. Surg..

[3] Armstrong D.G., Cohen K., Courric S., Bharara M., Marston W. (2011). Diabetic foot ulcers and vascular insufficiency: Our population has changed, but our methods have not. J. Diabetes Sci. Technol..

[4] Fu X.L., Ding H., Miao W.W., Mao C.X., Zhan M.Q., Chen H.L. (2019). Global recurrence rates in diabetic foot ulcers: A systematic review and meta-analysis. Diabetes Metab. Res. Rev..

[5] Schaper N.C., van Netten J.J., Apelqvist J., Bus S.A., Hinchliffe R.J., Lipsky B.A., Board I.E. (2020). Practical Guidelines on the prevention and management of diabetic foot disease (IWGDF 2019 update). Diabetes Metab. Res. Rev..

[6] Noor S., Khan R.U., Ahmad J. (2017). Understanding Diabetic Foot Infection and its Management. Diabetes Metab. Syndr..

[7] Johani K., Malone M., Jensen S., Gosbell I., Dickson H., Hu H., Vickery K. (2017). Microscopy visualisation confirms multi-species biofilms are ubiquitous in diabetic foot ulcers. Int. Wound J..

[8] Tawre M.S., Kamble E.E., Kumkar S.N., Mulani M.S., Pardesi K.R. (2021). Antibiofilm and antipersister activity of acetic acid against extensively drug resistant Pseudomonas aeruginosa PAW1. PLoS ONE.

[9] Vatan A., Saltoglu N., Yemisen M., Balkan I.I., Surme S., Demiray T., Mete B., Tabak F., Study Group C.r.D.F. (2018). Association between biofilm and multi/extensive drug resistance in diabetic foot infection. Int. J. Clin. Pract..

[10] Tacconelli E., Carrara E., Savoldi A., Harbarth S., Mendelson M., Monnet D.L., Pulcini C., Kahlmeter G., Kluytmans J., Carmeli Y. (2018). Discovery, research, and development of new antibiotics: The WHO priority list of antibiotic-resistant bacteria and tuberculosis. Lancet Infect. Dis..

[11] DeLeon S., Clinton A., Fowler H., Everett J., Horswill A.R., Rumbaugh K.P. (2014). Synergistic interactions of Pseudomonas aeruginosa and Staphylococcus aureus in an in vitro wound model. Infect. Immun..

[12] Mashburn L.M., Jett A.M., Akins D.R., Whiteley M. (2005). Staphylococcus aureus serves as an iron source for Pseudomonas aeruginosa during in vivo coculture. J. Bacteriol..

[13] Mitchell G., Séguin D.L., Asselin A.E., Déziel E., Cantin A.M., Frost E.H., Michaud S., Malouin F. (2010). Staphylococcus aureus sigma B-dependent emergence of small-colony variants and biofilm production following exposure to Pseudomonas aeruginosa 4-hydroxy-2-heptylquinoline-N-oxide. BMC Microbiol..

[14] Sharma S., Sahoo N., Bhunia A. (2016). Antimicrobial Peptides and their Pore/Ion Channel Properties in Neutralization of Pathogenic Microbes. Curr. Top. Med. Chem..

[15] Mann R., Holmes A., McNeilly O., Cavaliere R., Sotiriou G.A., Rice S.A., Gunawan C. (2021). Evolution of biofilm-forming pathogenic bacteria in the presence of nanoparticles and antibiotic: Adaptation phenomena and cross-resistance. J. Nanobiotechnol..

[16] Sindeldecker D., Stoodley P. (2021). The many antibiotic resistance and tolerance strategies of. Biofilm.

[17] Liu Y., She P., Xu L., Chen L., Li Y., Liu S., Li Z., Hussain Z., Wu Y. (2021). Antimicrobial, Antibiofilm, and Anti-persister Activities of Penfluridol Against. Front. Microbiol..

[18] Dumville J.C., Lipsky B.A., Hoey C., Cruciani M., Fiscon M., Xia J. (2017). Topical antimicrobial agents for treating foot ulcers in people with diabetes. Cochrane Database Syst. Rev..

[19] Boulton A.J.M., Armstrong D.G., Hardman M.J., Malone M., Embil J.M., Attinger C.E., Lipsky B.A., Aragón-Sánchez J., Li H.K., Schultz G. (2020). Diagnosis and Management of Diabetic Foot Infections. Compendia.

[20] Suner S.S., Sahiner M., Ayyala R.S., Bhethanabotla V.R., Sahiner N. (2021). Versatile Fluorescent Carbon Dots from Citric Acid and Cysteine with Antimicrobial, Anti-biofilm, Antioxidant, and AChE Enzyme Inhibition Capabilities. J. Fluoresc..

[21] Lei J., Sun L., Huang S., Zhu C., Li P., He J., Mackey V., Coy D.H., He Q. (2019). The antimicrobial peptides and their potential clinical applications. Am. J. Transl. Res..

[22] Serrano I., Oliveira M., Serrano I. (2015). Antimicrobial Peptides. Frontiers in Antimicrobial Agents—The Challenging of Antibiotic Resistance in the Development of New Therapeutics.

[23] Cotter P.D., Ross R.P., Hill C. (2013). Bacteriocins—A viable alternative to antibiotics?. Nat. Rev. Microbiol..

[24] Shin J.M., Gwak J.W., Kamarajan P., Fenno J.C., Rickard A.H., Kapila Y.L. (2016). Biomedical applications of nisin. J. Appl. Microbiol..

[25] Mulders J.W., Boerrigter I.J., Rollema H.S., Siezen R.J., de Vos W.M. (1991). Identification and characterization of the lantibiotic nisin Z, a natural nisin variant. Eur. J. Biochem..

[26] Field D., O’ Connor R., Cotter P.D., Ross R.P., Hill C. (2016). In Vitro Activities of Nisin and Nisin Derivatives Alone and In Combination with Antibiotics against Staphylococcus Biofilms. Front. Microbiol..

[27] Delvesbroughton J. (1993). The use of EDTA to enhance the efficacy of nisin towards gram-negative bacteria. Int. Biodeterior. Biodegrad..

[28] Leive L. (1965). Release of lipopolysaccharide by EDTA treatment of E. coli. Biochem. Biophys. Res. Commun..

[29] Thombare N., Jha U., Mishra S., Siddiqui M.Z. (2016). Guar gum as a promising starting material for diverse applications: A review. Int. J. Biol. Macromol..

[30] Verma D., Sharma S.K. (2021). Recent advances in guar gum based drug delivery systems and their administrative routes. Int. J. Biol. Macromol..

[31] Santos R., Gomes D., Macedo H., Barros D., Tibério C., Veiga A.S., Tavares L., Castanho M., Oliveira M. (2016). Guar gum as a new antimicrobial peptide delivery system against diabetic foot ulcers Staphylococcus aureus isolates. J. Med. Microbiol..

[32] Soares R.S., Santos R., Cunha E., Tavares L., Trindade A., Oliveira M. (2020). Influence of Storage on the Antimicrobial and Cytotoxic Activities of a Nisin-biogel with Potential to be Applied to Diabetic Foot Infections Treatment. Antibiotics.

[33] Mendes J.J., Marques-Costa A., Vilela C., Neves J., Candeias N., Cavaco-Silva P., Melo-Cristino J. (2012). Clinical and bacteriological survey of diabetic foot infections in Lisbon. Diabetes Res. Clin. Pract..

[34] Mottola C., Semedo-Lemsaddek T., Mendes J.J., Melo-Cristino J., Tavares L., Cavaco-Silva P., Oliveira M. (2016). Molecular typing, virulence traits and antimicrobial resistance of diabetic foot staphylococci. J. Biomed. Sci..

[35] Mottola C., Mendes J.J., Cristino J.M., Cavaco-Silva P., Tavares L., Oliveira M. (2016). Polymicrobial biofilms by diabetic foot clinical isolates. Folia. Microbiol..

[36] Mottola C., Matias C.S., Mendes J.J., Melo-Cristino J., Tavares L., Cavaco-Silva P., Oliveira M. (2016). Susceptibility patterns of Staphylococcus aureus biofilms in diabetic foot infections. BMC Microbiol..

[37] Wiegand I., Hilpert K., Hancock R.E. (2008). Agar and broth dilution methods to determine the minimal inhibitory concentration (MIC) of antimicrobial substances. Nat. Protoc..

[38] Ceri H., Olson M.E., Stremick C., Read R.R., Morck D., Buret A. (1999). The Calgary Biofilm Device: New technology for rapid determination of antibiotic susceptibilities of bacterial biofilms. J. Clin. Microbiol..

[39] Kaplan J.B. (2011). Antibiotic-induced biofilm formation. Int. J. Artif. Organs.

[40] ANSI, AAMI, ISO (2009). Biological Evaluation of Medical Devices—Part 5: Tests for Cytoxicity: In Vitro Methods.

[41] French G.L. (2006). Bactericidal agents in the treatment of MRSA infections--the potential role of daptomycin. J. Antimicrob. Chemother..

[42] Alford M.A., Mann S., Akhoundsadegh N., Hancock R.E.W. (2022). Competition between Pseudomonas aeruginosa and Staphylococcus aureus is dependent on intercellular signaling and regulated by the NtrBC two-component system. Sci. Rep..

[43] Kamarajan P., Hayami T., Matte B., Liu Y., Danciu T., Ramamoorthy A., Worden F., Kapila S., Kapila Y. (2015). Nisin ZP, a Bacteriocin and Food Preservative, Inhibits Head and Neck Cancer Tumorigenesis and Prolongs Survival. PLoS ONE.

[44] Bácskay I., Nemes D., Fenyvesi F., Váradi J., Vasvári G., Fehér P., Vecsernyés M., Ujhelyi Z. (2017). Role of Cytotoxicity Experiments in Pharmaceutical Development. Cytotoxicity.

[45] Lanigan R.S., Yamarik T.A. (2002). Final report on the safety assessment of EDTA, calcium disodium EDTA, diammonium EDTA, dipotassium EDTA, disodium EDTA, TEA-EDTA, tetrasodium EDTA, tripotassium EDTA, trisodium EDTA, HEDTA, and trisodium HEDTA. Int. J. Toxicol..

[46] Al-Hity A., Ramaesh K., Lockington D. (2018). EDTA chelation for symptomatic band keratopathy: Results and recurrence. Eye.

[47] Finnegan S., Percival S.L. (2015). EDTA: An Antimicrobial and Antibiofilm Agent for Use in Wound Care. Adv. Wound Care.

[48] George T., Brady M.F. (2022). Ethylenediaminetetraacetic Acid (EDTA). StatPearls.

